# Reframing Dementia Prevention Strategies Aligned with the WHO Global Action Plan: A Structured Narrative Review Focusing on Mild Behavioral Impairment

**DOI:** 10.3390/neurolint18010018

**Published:** 2026-01-16

**Authors:** Efthalia Angelopoulou, Sokratis Papageorgiou, John Papatriantafyllou

**Affiliations:** 1 1st Department of Neurology, Eginition Hospital, National and Kapodistrian University of Athens, 11528 Athens, Greece; sokpapa@med.uoa.gr (S.P.); jpapatriantafyllou@gmail.com (J.P.); 2 Third Age Day Care Center IASIS, 16562 Athens, Greece

**Keywords:** dementia prevention, mild behavioral impairment, prodromal dementia, preclinical dementia, Alzheimer’s disease, mild behavioral impairment checklist, dementia risk, neurodegenerative diseases, mild cognitive impairment

## Abstract

**Background/Objectives**: Dementia represents a growing public health challenge. The WHO Global Action Plan on the Public Health Response to Dementia emphasizes early detection, risk reduction, and innovation as key priorities. Mild Behavioral Impairment (MBI), defined as the emergence of persistent neuropsychiatric symptoms in older individuals, represents a potential marker of early neurodegeneration and possible window for early intervention. This review explores the role of MBI in dementia prevention, mapping current evidence within the WHO Global Action Plan framework. **Methods**: A comprehensive search was performed in PubMed, Scopus, and the official WHO website, during 1 September 2025–10 November 2025, without time restrictions. Eligible sources included original clinical studies, reviews, and policy documents addressing MBI, dementia prevention, and public health. Data were thematically synthesized according to the seven objectives of WHO: (1) dementia as a public health priority, (2) dementia awareness and friendliness, (3) dementia risk reduction, (4) dementia diagnosis, treatment, care and support, (5) support for dementia carers, (6) information systems for dementia, and (7) dementia research and innovation. **Results**: Accumulating evidence indicates that MBI assessment can capture early behavioral manifestations of neurodegenerative and other forms of dementia, correlating with fluid, neuroimaging and genetic biomarkers. Integrating MBI screening through the easy-to-administer MBI Checklist (MBI-C) into clinical and community-based care, including telemedicine pathways and research, may enhance early identification and personalized interventions, enrich the pool for clinical trials, and facilitate research in biomarker and therapy. MBI-related research further supports its integration in remote digital monitoring and population-based prevention. **Conclusions**: Embedding MBI-informed screening and interventions into national dementia strategies aligns with WHO objectives for early, equitable and scalable prevention and brain health.

## 1. Introduction

**Figure 1:**
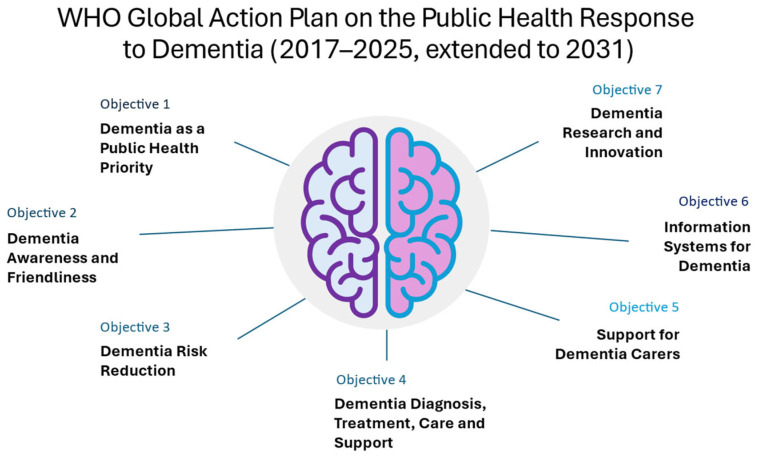
The seven objectives of the World Health Organization (WHO) introduced the Global Action Plan on the Public Health Response to Dementia (2017–2025, extended to 2031).

Dementia is one of the most pressing public health challenges of the 21st century, affecting more than 55 million people globally, and imposing an enormous socioeconomic burden on individuals, families, and healthcare systems [1]. As the population ages, the prevalence of dementia is estimated to triple by 2050, highlighting the urgent need for effective prevention and early intervention strategies [2]. In this context, the World Health Organization (WHO) introduced the Global Action Plan on the Public Health Response to Dementia (2017–2025, extended to 2031), a landmark policy framework aiming to address the impact of dementia through seven strategic objectives encompassing public awareness, risk reduction, early detection and intervention, care improvement, and research innovation (Figure 1) [2].

One of the key principles of the WHO Global Action Plan is the recognition that dementia prevention should begin before the onset of cognitive symptoms [2]. Emphasis is placed on the identification of high-risk individuals and early interventions aiming to delay or prevent cognitive decline. Traditionally, research and prevention efforts have focused on cognitive constructs such as mild cognitive impairment (MCI) and subjective cognitive decline (SCD), as early manifestations of neurodegeneration. However, accumulating evidence suggests that later-life emergent neuropsychiatric (NPS) symptoms, conceptualized as Mild Behavioral Impairment (MBI), may constitute an even earlier clinical sign of Alzheimer’s disease (AD) and other types of dementia [3].

Preclinical dementia denotes the presence of underlying neuropathological changes, such as amyloid and tau accumulation, in individuals who are cognitively and functionally unimpaired and may be entirely asymptomatic. In contrast, prodromal dementia refers to a symptomatic stage in which subtle clinical changes are present, traditionally characterized by MCI, but increasingly recognized to include non-cognitive manifestations. Within this framework, MBI is conceptualized as a behavioral prodrome that may occur in cognitively unimpaired individuals or alongside early cognitive changes, bridging the transition between preclinical pathology and clinically overt dementia [3].

**Figure 2:**
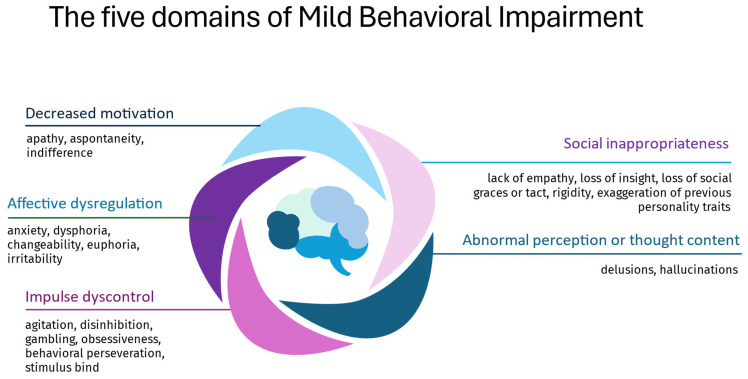
The five domains of Mild Behavioral Impairment (MBI).

The International Society to Advance Alzheimer’s Research and Treatment-Alzheimer’s Association (ISTAART-AA) has defined MBI as the onset of sustained (≥6 months) NPS in older individuals (≥50 years) without dementia that represents a change from their longstanding behavior [3]. According to the ISTAART-AA MBI criteria, these NPS should not be explained by other psychiatric disorders, and they are organized into five domains: apathy, affective/emotional dysregulation, impulse dyscontrol, social inappropriateness, and abnormal perception/thought content (Figure 2).

MBI has been associated with AD fluid and neuroimaging biomarkers, further supporting that it may be the earliest clinical manifestation of the underlying neurodegenerative process [4]. The MBI Checklist (MBI-C) is a standardized tool for the assessment of NPS in the context of MBI, in both clinical and research settings [5]. It is freely available and can be administered by non-specialist clinicians and caregivers, representing a feasible and low-cost approach for large-scale screening in the community [5].

Despite growing evidence, the role of MBI in global dementia prevention strategies has not yet been systematically contextualized within public health policy frameworks. Aligning MBI research with the WHO Global Action Plan offers a unique opportunity to strengthen early detection, foster innovation in service delivery, and support person-centered prevention initiatives. This is particularly relevant for countries where access to biomarker testing is limited, and behavioral assessment may provide a more equitable and scalable entry point for identifying at-risk populations and broaden the window for interventions.

In this structured narrative review, we discuss the role of MBI in relation to dementia prevention strategies, mapping current evidence onto the seven objectives of the WHO Global Action Plan on Dementia (2017–2025, extended to 2031). By synthesizing findings from clinical, epidemiological, and policy research, this review seeks to highlight how integrating MBI into dementia prevention frameworks can enhance early identification, improve caregiver and community engagement, and advance innovation in dementia care and public health planning.

## 2. Materials and Methods

The methodological principles of a structured narrative review, guided by the Scale for the Assessment of Narrative Review Articles (SANRA) recommendations [6]. This approach was selected to allow a transparent yet flexible synthesis of heterogeneous clinical, epidemiological, biomarker, and policy-related evidence, which is not readily amenable to meta-analytic techniques. A comprehensive search was conducted in three sources: PubMed, Scopus and the official WHO website (https://www.who.int/) during 1 September 2025–10 November 2025, without restrictions on publication year. The following keywords in different combinations were used with Boolean operators (AND/OR): “mild behavioral impairment”, “MBI Checklist”, “MBI-C”, “neuropsychiatric symptoms”, “prodromal”, “preclinical”, “dementia”, “prevention”, “risk reduction”, “public health,” and “WHO Global Action Plan”.

Following the initial search, all retrieved records were screened at the title and abstract level to assess relevance. Full-text screening was subsequently performed for potentially eligible articles. In addition, the reference lists of all included articles were manually reviewed to identify further relevant publications not captured in the database search. Eligible sources included original clinical studies (epidemiological, biomarker, neuroimaging, or clinical cohort studies), systematic or narrative reviews, and policy or strategic documents addressing MBI in the context of dementia prevention, early detection or public health frameworks. Only articles published in English were included. Studies were excluded if they focused exclusively on established dementia without reference to pre-dementia stages, addressed NPS unrelated to MBI, or lacked relevance to dementia prevention or public health perspectives. However, for discussion purposes, articles related to dementia or dementia-related NPS were also read and referred to when appropriate.

Data extraction focused on study characteristics, population, MBI definition and assessment method, key outcomes, and relevance to dementia prevention or public health. The included evidence was thematically synthesized, mapping findings onto the seven objectives of the WHO Global Action Plan on the Public Health Response to Dementia. Although several studies could reasonably contribute to more than one objective, each piece of evidence was assigned to the single objective considered most conceptually relevant.

Given the rapidly expanding literature on MBI, and the presence of multiple prior reviews, particular emphasis was placed on studies published within the last three years, to ensure contemporary relevance and alignment with current scientific and policy developments. Nevertheless, earlier studies were included where necessary to support conceptual definitions and foundational evidence, particularly regarding MBI diagnostic criteria, assessment tools, and core aspects related to dementia prevention.

## 3. Results

**Figure 3:**
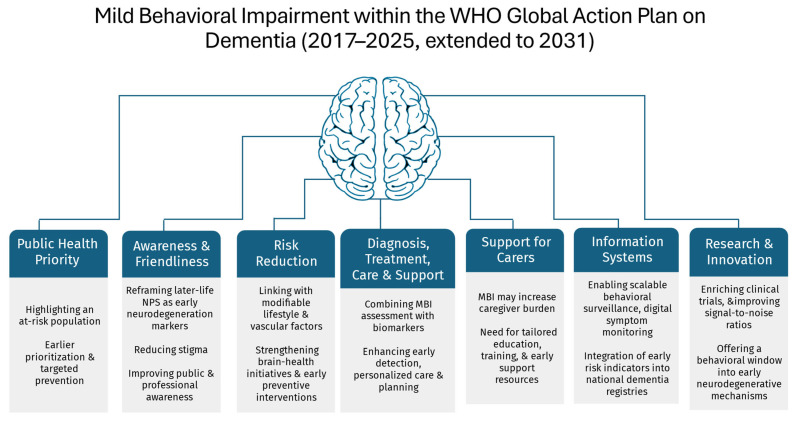
MBI within the WHO Global Action Plan on Dementia (2017–2025, extended to 2031).

This review synthesizes recent evidence on MBI through the lens of the WHO Global Action Plan on Dementia, highlighting its relevance for early detection, prevention, and public-health preparedness. The key insights are summarized below (Table 1, Figure 3).

### 3.1. WHO Global Action Plan on the Public Health Response to Dementia: Overview

The WHO’s Global Action Plan on the Public Health Response to Dementia 2017–2025 represents a coordinated international framework for addressing dementia as a global health priority [2,36]. It was unanimously approved during the 70th World Health Assembly, on 29 May 2017, in Geneva.

The plan highlights the urgent need for the development of ambitious and practical strategies by all governments, to meet seven main targets and action areas: (1) dementia as a public health priority, (2) dementia awareness and friendliness, (3) dementia risk reduction, (4) dementia diagnosis, treatment, care and support, (5) support for dementia carers, (6) information systems for dementia, and (7) dementia research and innovation. It is grounded in a rights-based approach that emphasizes autonomy, equity, and social inclusion, while simultaneously advancing a public-health agenda focused on prevention and early detection.

Despite variations in national dementia strategies, the Global Action Plan provides governments with a structured blueprint to guide policy development, improve service delivery, and strengthen public understanding of dementia. Importantly, its successful implementation will depend on sustained political commitment, adequate funding, and meaningful engagement of civil society, particularly in low- and middle-income countries where resources are limited. However, it has been recently shown that most countries, particularly low- and middle-income nations, are far from achieving the global action plan targets, despite carrying the greatest share of the dementia burden. Consequently, in May 2025, WHO Member States agreed to extend the plan’s timeline to 2031 to allow continued implementation and monitoring.

### 3.2. Dementia as a Public Health Priority (Objective 1)

Epidemiological evidence consistently show that MBI is associated with a higher risk of incident cognitive decline and dementia [7,8,9,10,11,12,13,14,15]. It has been shown that approximately two third of individuals with MCI or dementia had developed NPS before the onset of cognitive symptoms, with irritability and depression being the most common ones [37].

A recent systematic review and meta-analysis including over 5000 participants reported that approximately 52% of people with MCI also meet criteria for MBI [38], underscoring its substantial prevalence within the pre-dementia population. In MCI, the most commonly affected MBI domains are impulse dyscontrol, affective dysregulation, and decreased motivation [30,39]. Evidence from the NACC indicates that older individuals with normal cognition or MCI meeting MBI criteria experience faster progression to dementia, compared to those with NPS not meeting MBI criteria or no NPS [40]. This time-dependent, accelerated decline highlights MBI as a high-risk subgroup within the aging population, with a clear need to early recognize MBI as a pivotal public health issue.

A broader societal perspective further underscores the public health relevance of MBI. Historical and contemporary analyses of political leadership highlight that later-life cognitive and behavioral changes, including MCI and MBI, have influenced high-stakes decision-making, international negotiations, and geopolitical stability, such as the emergence of Nazi in 1930s in Germany, the collapse of communist regimes, the Arab Spring and others [41]. Such cases illustrate how unrecognized behavioral changes in ageing leaders may carry substantial societal risks, reinforcing the need for public awareness, destigmatization, and systematic approaches to early detection. Incorporating MBI into public health agenda therefore supports a more informed, ethical, and safety-oriented understanding of cognitive aging at the population level.

Hence, MBI may constitute a new public health construct bridging mental and cognitive health, supporting the public health view of dementia as a continuum starting with late-life behavioral change.

### 3.3. Dementia Awareness and Friendliness (Objective 2)

Increasing awareness and preparedness among healthcare professionals is essential for identifying MBI. As highlighted in current clinical practice models, primary care providers and interdisciplinary teams play a pivotal role in recognizing early cognitive and behavioral shifts, yet these signs may be overlooked, misattributed to normal aging, or overshadowed by time constraints and limited familiarity with the syndrome of MBI and its assessment [16]. Enhancing healthcare professionals’ knowledge and confidence toward integrating MBI screening into routine pathways could bridge a diagnostic gap and promote timely referrals.

Although research on stigma specifically associated with MBI is still limited, it is possible that individuals exhibiting later-life NPS may encounter experiences similar to those described in MCI, including misinterpretation of symptoms, fear of future cognitive decline, and negative social reactions [42]. Because MBI may reflect behavioral changes that are often subtle, poorly understood, and easily attributed to personality flaws or mental illness rather than early neurodegeneration, affected individuals may face heightened risk of misunderstanding, social withdrawal, and internalized stigma [42]. In parallel with findings in MCI, the potential anticipation of dementia, or the vague perception by others that “something is wrong”, may amplify stress, reduce help-seeking behavior, and compromise quality of life [42]. Recognizing and addressing stigma around behavioral symptoms is therefore essential, both to improve patient and caregiver experience and to support the timely identification of individuals at increased risk for dementia.

### 3.4. Dementia Risk Reduction (Objective 3)

Risk reduction is a central pillar of the WHO public health response to dementia. The 2019 WHO Guidelines on risk reduction of cognitive decline and dementia synthesize evidence that multiple modifiable factors, including cardiovascular risk control, physical activity, diet, smoking, alcohol use, and social and cognitive engagement, can be targeted across the life course to lower dementia risk [43]. Accumulating epidemiological evidence links MBI to a higher risk of cognitive decline and dementia [17,18,19,20,21]. In a large recent study, ΜΒΙ was linked to AD pathology five years later, while this relationship was more strong in cognitively intact participants at baseline [44]. In this context, MBI offers a potential complementary “neurobehavioral axis” for prevention, highlighting emergent later-life NPS as a clinically accessible target for risk stratification and early intervention.

It is well-known that female sex is associated with a higher risk for dementia. In this context, sex may modify the relationship between MBI and cognitive decline. In a large dementia-free cohort (*n* = 8181), males showed slightly higher MBI prevalence and stronger associations between several MBI domains and both baseline cognitive performance and subsequent cognitive decline, whereas females displayed a unique link between affective dysregulation and decline especially in verbal reasoning [45].

In another study in postmenopausal women, greater menopausal symptom burden was associated with both poorer cognitive function and increased MBI severity, while hormone therapy use was linked to lower MBI-C total score [46]. These findings suggest that sex-specific behavioral profiles may shape dementia risk and the menopausal transition may represent a critical window for dementia prevention, reinforcing the need for tailored prevention strategies that consider sex as an effect modifier in mid- and late-life behavioral changes.

Growing evidence indicates that dementia prevention must begin long before older adulthood [47]. A recent study showed that individuals with more severe adverse childhood experiences (ACEs) may exhibit higher MBI-C scores and poorer executive functioning in later life, independent of psychiatric history [48]. This suggests that early-life adversity may create long-term vulnerability to neurobehavioral dysregulation and cognitive impairment, reinforcing the principle that dementia prevention should span the entire life course.

Cognitive reserve refers to the accumulation of enriching experiences during the lifetime, including higher education, cognitively demanding occupations, and engagement in stimulating activities, which are considered to enhance resilience to neuropathology. Recent evidence from the CAN-PROTECT cohort shows that higher cognitive reserve is associated not only with better cognitive performance but also with lower likelihood of MBI and its severity, particularly in the domains of impulse dyscontrol and social inappropriateness [49]. These findings highlight cognitive reserve as a potential protective factor against early neurobehavioral change, reinforcing its importance as a target for lifelong dementia risk-reduction strategies.

It has been recently demonstrated that Black individuals with dementia might display more risk factors, worse cognitive function, greater functional decline and more severe NPS, compared to White individuals [50]. In this context, affective dysregulation has been linked to higher dementia risk, while this association was stronger in Black than in White participants, emphasizing that MBI-related risk may be also shaped by social and contextual factors [51]. These findings highlight the need for culturally sensitive risk assessment and targeted prevention strategies that consider MBI domains in under-represented populations.

Social isolation is one of the fourteen modifiable risk factors of dementia, according to the 2024 report of the Lancet standing Commission [47]. In turn, social support has been recently shown to be inversely correlated with MBI, through enhanced psychological resilience, with anxiety levels and sleep quality further moderating this relationship [52]. In the same vein, findings from Thai community-dwelling older adults indicate that greater social support is associated with lower MBI burden [53], underscoring the central role of social connectedness in mitigating early behavioral vulnerability.

Physical inactivity presents another pivotal modifiable dementia risk factor [47]. Importantly, moderate-to-vigorous physical activity has been associated with reduced MBI burden and better cognitive performance in older adults, with MBI partially mediating this relationship [53]. Furthermore, evidence from the CAN-PROTECT cohort shows that higher levels of cardiovascular activity are linked to reduced severity of MBI symptoms, whereas sustained physical labor is related to higher MBI severity, suggesting that not all activity types confer equal neuroprotective effects [54]. These results support the hypothesis that some lifestyle factors promoting brain health, including physical activity, may exert part of their beneficial effect by attenuating emergent behavioral changes characteristic of MBI.

Further evidence highlights that fear of falling, which is linked to reduced mobility and physical inactivity, is associated with MBI-related social behavior changes [55]. Moreover, dual-task gait cost, another non-cognitive dementia marker, has been shown to correlate with greater global MBI burden, with executive dysfunction mediating this association [56]. This reinforces the shared motor-behavioral pathway in early neurodegeneration and suggests that gait assessments may offer additional value in identifying individuals with MBI.

In addition to physical inactivity, high LDL cholesterol, diabetes, smoking, hypertension and obesity in midlife constitute cardiovascular factors increasing the risk of dementia [47]. Elevated fasting glucose levels have been correlated with MBI [57], and in individuals with MCI, diabetes mellitus is associated with both higher prevalence and greater severity of MBI [58], reinforcing diabetes as a modifiable factor linking vascular burden with emergent behavioral changes.

Traumatic brain injury (TBI) is another modifiable risk factor for dementia [47]. However, recent evidence suggests that a history of TBI is not associated with meaningful differences in the cross-sectional relationship between MCI and MBI symptoms, nor with differences in the prospective relationship between MCI and incident dementia [59]. These results imply that MBI retains its prognostic relevance even in individuals with a history of a prior head injury. Moreover, prior head injury, particularly two or more injuries, has been related to increased MBI burden, mainly in the impulse dyscontrol and affective dysregulation domains [60], whereas more severe TBI, defined as loss of consciousness of more than 5 min, has been linked to social inappropriateness [61]. In another study, TBI was particularly associated with apathy in a pre-dementia population [62]. Suspected mild TBI has also been associated with a higher likelihood of both SCD and MBI in cognitively unimpaired older adults, with these early cognitive-behavioral changes mediating the relationship between TBI and poorer everyday functioning [63]. This suggests that while TBI may not modify the prognostic value of MBI within MCI, it may nevertheless contribute to later-life vulnerability by potentially increasing the likelihood of emergent behavioral and cognitive symptoms in the preclinical or prodromal stages.

Hearing loss is a key modifiable risk factor for dementia [47]. Interestingly, subjective hearing disability has been linked to greater overall MBI burden, particularly in apathy and affective dysregulation domains [12]. Furthermore, recent evidence links untreated hearing loss to a higher likelihood of MBI and specific domains, including affective dysregulation, impulse dyscontrol, and social inappropriateness [64,65]. Importantly, treated hearing loss, such as through hearing aids, was not associated with higher MBI burden compared to those without hearing loss, highlighting the preventive potential of timely treatment [64,65]. Longitudinal bidirectional associations were also found between hearing loss and MBI, further supporting the integration of hearing assessment and rehabilitation into comprehensive risk-reduction strategies [64,65]. Hence, early identification and management of sensory deficits may offer a practical avenue targeting MBI and dementia risk.

Sleep disturbance is an additional modifiable target within dementia risk reduction, although not included in the Lancet Commission 2024 update [47]. Notably, MBI and sleep disturbance show a potential bidirectional relationship, and their co-occurrence has been associated with more than a twofold risk of dementia, compared to sleep abnormalities alone, highlighting the need to integrate systematic assessment and management of sleep into preventive strategies [66].

Frailty represents another important condition that intersects with dementia risk and MBI. Recent evidence from the Comprehensive Assessment of Neurodegeneration and Dementia (COMPASS-ND) cohort shows that greater frailty burden is related to MBI and its severity particularly in men [67]. In accordance, MBI has been associated with a higher likelihood of frailty in another study [21], suggesting that MBI screening may be especially informative in frail older adults regarding dementia prevention.

The WHO’s Optimizing Brain Health across the Life Course position paper aligns closely with risk-reduction strategies in dementia, emphasizing early interventions across physical health, social connection, learning and safe environments [68]. Hence, the incorporation of MBI assessment and management in dementia prevention targets concurrently WHO’s mission towards brain health.

Collectively, these findings align with the observation that some MBI symptoms partially overlap with modifiable dementia risk factors, such as apathy with physical inactivity and dysphoria with social withdrawal. Consequently, early lifestyle or behavioral interventions may simultaneously reduce MBI burden and help counteract dementia risk, thereby constituting both primary and secondary preventive approaches.

### 3.5. Dementia Diagnosis, Treatment, Care and Support (Objective 4)

Early identification of dementia-related clinical change is a central pillar of Objective 4, and growing evidence supports the early integration of MBI into diagnostic and care frameworks throughout the continuum of neurodegeneration and other dementia-related diseases.

From a clinical point of view, MBI may signal neurodegeneration earlier than cognitive screening tools. According to a recent study, MBI symptoms were associated with cognitive performance only once MoCA scores had fallen below a certain threshold, and the cognitive-behavioral relationship was stronger with poorer cognitive performance. These results imply that subtle cognitive deficits may remain undetected while behavioral changes are already evident [69]. MBI has been linked to more rapid decline in working memory and attention even in cognitively intact older adults [70]. A recent meta-analysis demonstrated that MBI has been consistently related to poorer cognitive performance in both global cognitive functioning and specific domains, including memory, and executive functions [71]. However, this inverse relationship was stronger when assessing the cognitive domains individually compared to a global cognitive evaluation [71]. These findings suggest that MBI may capture subtle, early cognitive changes possibly not detectable through traditional global screening measures, necessitating a detailed, comprehensive neuropsychological evaluation. It could be also proposed that MBI assessment might be a more sensitive screening tool for dementia risk detection compared to MMSE or MOCA, although further evidence is needed to test this hypothesis.

Concerning the different MBI symptoms, in a large cohort of community-dwelling older adults, decreased motivation was the only MBI domain that independently predicted incident dementia after 4.5 years [72]. In another study, the factors that most strongly predicted incident cognitive impairment were MBI overall, followed by the domains of impulse dyscontrol and affective dysregulation [73]. Furthermore, in amnestic MCI, the presence of multiple MBI domains, particularly apathy, affective dysregulation, and impulse dyscontrol, may increase the risk of progression to AD, compared to affective dysregulation alone [74]. This reinforces the potential utility of MBI domain-level assessment in identifying individuals who warrant closer monitoring, especially those with apathy or symptoms from multiple MBI domains.

Accumulating evidence highlights the diagnostic relevance of MBI within the updated 2024 NIA-AA research framework for the diagnosis of AD, which recognizes behavioral change as an early clinical indicator [75]. In a large Alzheimer’s Disease Neuroimaging Initiative (ADNI) cohort, individuals meeting MBI criteria displayed increased likelihood of CSF Aβ42 and p-tau181 positivity [26]. In accordance, MBI has been linked to lower CSF Aβ42, Aβ42/40 ratio, as well as higher CSF p-tau, t-tau, p-tau/Aβ42, and t-tau/Aβ42 ratio in another study [7]. MBI presence and/or severity has also been correlated with the blood levels of Aβ42 [76], p-tau217 [76,77] in older individuals with normal cognition or MCI, as well as overall MBI [78] and MBI psychosis [79] with plasma p-tau181. Longitudinal evidence shows that increased plasma p-tau181/Aβ42 ratio, and p-tau181 at baseline were associated with a higher risk of MBI incidence [80].

These findings suggest that MBI is closely aligned with the NIA-AA Core 1 biomarkers (amyloid and phosphorylated tau pathology defining the AD continuum) [75], strengthening its potential role in early detection and guiding timely diagnostic evaluation. Placing MBI within the updated NIA-AA framework underscores its relevance as an early bridge between clinical presentation and underlying neuropathological change.

Recent tau-PET Braak staging work further reinforces the diagnostic significance of MBI as an early clinical correlate of tau pathology [81]. Across the AD continuum, higher Braak stages were associated with greater MBI-C severity at baseline, while stages V–VI predicted an annual elevation of MBI burden [81]. Hence, tau accumulation may clinically manifest via increasing NPS, and integrating MBI assessment with tau-PET might enhance diagnostic precision within the NIA-AA framework. Importantly, MBI has been related to tau pathology independently of episodic memory deficits [82]. Consistent evidence from an ADNI tau-PET study further shows that, among Aβ-positive individuals with normal cognition or MCI, MBI is associated with greater tau tracer uptake in Braak I and III cortical regions [83], suggesting that MBI could help identify individuals with early AD-related tau deposition.

Furthermore, MBI has been associated with amyloid pathology in amyloid PET [31,84], with this burden at least partially mediating the effect of MBI on global cognition and several domains including memory, language and executive function [31]. Longitudinal evidence demonstrated that in addition to the higher risk of cognitive decline, MBI was linked to more rapid increase in amyloid burden [31]. Hence it could be proposed that MBI could possibly constitute a marker of an accelerated disease course in the pre-dementia older population.

In addition to fluid or PET AD biomarkers, recent neuropathological data supports the clinical relevance of MBI for identifying persons at risk for AD [18,44]. In a NACC autopsy sample (*n* = 1.016), MBI was associated with pathologically confirmed AD five years later and predicted faster progression to AD dementia versus no NPS [44]. This effect was particularly pronounced among individuals with normal cognition at baseline. Importantly, associations were not observed for transactive response DNA-binding protein 43 (TDP-43) or overall Lewy body disease (LBD) pathology, although limbic LBD moderated the MBI-incident AD relationship. This evidence highlights the potential of MBI for dementia risk stratification in cognitively intact individuals, especially for AD.

Emerging evidence suggests that MBI may not represent a single uniform construct but rather encompass distinct behavioral endophenotypes with differential prognostic value for dementia risk. A data-driven analysis based on the National Alzheimer’s Coordinating Center (NACC) data from 20,599 older individuals with normal cognition identified four NPS profiles with graded risk of progression to MCI or dementia: Low-All < High-Depression < High-Agitation/Anxiety/Irritability (hAAI) < High-All [22]. hAAI and High-All groups demonstrated a higher risk of cognitive decline compared to depressive symptoms alone, and hAAI group showed increased burden of amyloid and neuritic plaques, and more severe arteriosclerosis [22]. These findings suggest that MBI symptom clusters may hold diagnostic relevance by reflecting distinct underlying pathophysiological pathways. Recognizing these differential behavioral signatures could support earlier, mechanism-informed diagnostic decisions and guide individualized care planning.

Converging evidence indicates that pairing MBI with fluid biomarkers or APOE genotyping enhances early detection and biological profiling of dementia risk [23]. A longitudinal study showed that cognitively intact older individuals with emergent NPS and concurrent AD biomarker positivity (increased CSF phosphorylated tau181) exhibited a markedly greater rate of incident MCI, compared to participants without either risk factor [23]. Individuals with both APOE ε4 allele and emergent NPS also displayed higher risk of MCI compared to APOE ε4 carriers without NPS [23]. These findings support the idea that MBI-like later-life NPS are not only early clinical indicators of impending cognitive decline, but that their predictive value might be amplified when combined with underlying neurobiological evidence of AD pathology. Integrating behavioral assessment within the MBI framework together with fluid or imaging biomarkers may therefore enhance early risk stratification and more accurately forecast cognitive trajectories, with direct implications for prevention-oriented pathways and timing of diagnostic evaluation.

As it has been extensively reviewed elsewhere, from the AT(N) perspective, in addition to amyloid and tau, limited but growing evidence highlights the relationship between MBI with neurodegeneration markers, including CSF total tau or metabolic decline [85]. Together, these findings support the positioning of MBI within the AT(N) framework as early, non-cognitive clinical expressions of underlying AD biology.

Emerging evidence also links MBI with disease progression biomarkers. In this regard, longitudinal evidence has shown that the emergence of MBI was associated with increased neurofilament light (NfL) levels at baseline [85,86]. In a large ADNI cohort, MBI was associated with significantly faster increases in plasma NfL over two years, independent of age or cognitive diagnosis (normal cognition or MCI) [86]. In addition, new-onset MBI was associated with accelerated NfL rise [86]. These findings support MBI as a clinical proxy of early axonal injury, supporting its additional prognostic value regarding the rate of progression of the underlying neurodegenerative process.

An important aspect that should be also considered is the fact that informants’ characteristics may significantly affect the reporting of NPS in the context of MBI, thereby possibly influencing its assessment [87]. A large-scale analysis from the NACC (*n* > 26,000) show that female, younger, and spouse informants identified more NPS [87], highlighting the importance of considering study-partner characteristics in MBI assessment and diagnostic decision-making.

Personality factors may further refine the clinical interpretation of MBI. In a caregiver-rated study, low agreeableness was linked to impulse dyscontrol, higher neuroticism to affective/emotional dysregulation, and low conscientiousness to decreased motivation [88]. Importantly, these relationships were stronger in current compared to premorbid ratings for personality traits [88], suggesting that MBI symptoms are partly shaped by personality, thereby supporting a more person-centered diagnostic and care approach.

MBI has also been linked to subtle functional impairment in everyday life. In an ADNI cohort of amnestic MCI (aMCI) and cognitively normal older adults, individuals with MBI showed higher Functional Assessment Questionnaire (FAQ) scores compared to those without MBI, indicating greater difficulty with instrumental activities of daily living [24]. FAQ evaluates difficulties in daily activities such as tax management, bill payment, remembering appointments and medications, tracking current events, cooking, hobbies and traveling [89]. These findings raise the possibility that early, targeted interventions, such as occupational therapy and compensatory strategies focusing on higher-order activities could help maintain functional independence for longer in people with MBI. Thus, structured support and rehabilitation even at this early stage may be beneficial, reinforcing its relevance for care planning and prevention-focused clinical pathways.

Furthermore, recent data also link MBI with poorer health-related quality of life (HRQoL) in older individuals without dementia. In a study of individuals with subjective cognitive decline (SCD) and MBI, both conditions independently predicted poorer physical HRQoL [25]. Specific MBI domains showed differential effects: impulse dyscontrol related to lower physical HRQoL, while affective dysregulation to worse mental HRQoL [25]. In accordance, higher MBI-C scores were inversely correlated to quality of life in another study [90]. The MBI-C total score, as well as the domains of decreased motivation and emotional dysregulation were related to poorer quality of life in another study [91]. Hence, MBI carries a meaningful burden on everyday well-being before cognitive decline emerges, highlighting the need for early psychosocial and behavioral support within care pathways for at-risk older adults.

Across all AD stages, current guidelines emphasize that non-pharmacological interventions constitute the first-line approach to managing NPS, with pharmacological treatments reserved only after reversible contributors are addressed [92]. Randomized clinical trials have shown that physical exercise may contribute to improved cognitive performance in individuals with MCI [93,94]. A recent randomized clinical trial further demonstrated that MBI may be amenable to early, non-pharmacological intervention [95]. A 7-week program combining moderate-intensity traditional Thai folk dance with cognitive stimulation led to significant reductions in MBI symptoms, alongside improvements in quality of life, and cognitive performance [95]. Another study demonstrated that a 13-week educative empowerment-based psycho-behavioral program on knowledge enhancement, cognitive coping and stress adaptation could exert beneficial effects on cognition, MBI symptoms and well-being in individuals with MCI [96]. These findings reinforce the potential clinical utility of early, multidomain behavioral interventions in mitigating early dementia-related changes and supporting well-being in older adults with MBI.

Clinical case-based evidence also reinforces how MBI can present with complex behavioral disturbances that require multi-disciplinary care. A representative example comes from a case report describing an older adult living in extreme domestic squalor with Diogenes-like behaviors, where refusal of help, hoarding, and profound motivation loss hindered intervention [97]. These later-life manifestations were ultimately interpreted as consistent with MBI, and improvement was achieved only through long-term, coordinated multidisciplinary involvement [97]. Such cases highlight the importance of early recognition of MBI to guide appropriate, sustained clinical management.

In summary, converging evidence positions MBI as a clinically meaningful precursor of neurodegenerative disease, detectable well before cognitive or functional thresholds are crossed. By integrating MBI assessment into routine diagnostic pathways, alongside biomarker evaluation and individualized non-pharmacological strategies, healthcare systems may enable earlier, more equitable, and more person-centered care.

### 3.6. Support for Dementia Carers (Objective 5)

Caregiver burden is increasingly recognized as a clinically meaningful consequence of emergent NPS in the early stages of neurodegenerative diseases. Evidence from a memory-clinic cohort has shown that MBI is associated with approximately a threefold higher caregiver burden in individuals with SCD or MCI, evaluated by the Zarit caregiver burden scale, independent of demographic factors and cognitive status [27]. These findings underscore that MBI symptoms have significant functional and psychosocial impact, reinforcing the need for early identification and support for caregivers as part of comprehensive care plan.

Based on the above evidence, there is a growing need for caregiver-oriented educational tools that address behavioral changes occurring before overt cognitive decline. While WHO’s iSupport program provides structured, evidence-based guidance for caregivers of people with established dementia [98], an updated or complementary version tailored to the early stages would fill an important gap. Incorporating modules on early NPS in the context of MBI could equip family members with the skills to recognize subtle behavioral shifts, seek timely assessment, and manage emerging challenges effectively.

Such an expansion would align with the Global Action Plan’s emphasis on strengthening carer support across the entire continuum of dementia. Given also the cross-national variability in caregiving norms, stigma, and health system structures, each country will need to culturally adapt any supportive material for carers [99]. Tailoring guidance to local beliefs, languages, and caregiving practices is essential to ensure relevance, acceptability, and real-world usability [99]. Nationally adapted versions of such caregiver tools could substantially enhance engagement and effectiveness across diverse settings.

### 3.7. Information Systems for Dementia (Objective 6)

Recent validation studies demonstrate that the MBI-C offers good diagnostic utility across the pre-dementia spectrum, with cut-offs of 6.5 in MCI and 8.5 in SCD with optimal sensitivity and specificity [28,29]. Phone-administered informant ratings reliably detected subtle NPS in both groups, and MBI-C scores showed expected correlations with NPI-Q, depressive symptoms, subjective complaints, and functional measures [28,29]. These findings support the feasibility of scalable, low-burden MBI screening in community settings.

From an information-systems perspective, the MBI-C offers a scalable, low-cost tool that can be deployed in digital platforms, primary care networks, or telemedicine systems to capture early behavioral changes [100]. Unlike extensive neuropsychological testing or specialized biomarker assessments, the MBI-C can be self- or informant-completed remotely, enabling large-scale, data-driven monitoring of at-risk populations and enhancing early-stage case finding. In this regard, the inclusion of MBI indicators in national registries and the WHO Global Dementia Observatory could enhance surveillance of preclinical and prodromal dementia and improve risk-stratification models.

Emerging digital approaches further expand the research landscape in early dementia detection. Remote, unsupervised assessment through platforms such as the Brain Health Registry has demonstrated that informant-rated MBI-C correlates with objective deficits in executive function and memory, confirming the feasibility of online behavioral screening [101]. These scalable digital tools align with global priorities to accelerate innovation, broaden access to early risk assessment, and support large-scale research infrastructures.

Beyond current clinical applications, emerging work highlights that MBI may also serve as a key behavioral feature in next-generation digital prediction models. A recent study protocol proposes the integration of MBI with multimodal markers, such as mobility, sleep/wake patterns, and social interaction via passive wearables, as well as ecological momentary assessments via smartphones, into machine-learning frameworks designed to predict functional decline in older individuals with SCD or MCI [102].

In addition to these developments, recent longitudinal work using wrist-worn sensors and deep-learning–derived digital biomarkers has shown that physiological features, such as heart-rate variability and skin conductance, may predict daily MBI severity in older adults with MCI [103]. Measuring behavioral changes in real-world and incorporate MBI in remote assessment platforms could enrich digital phenotyping and enhance precise risk stratification.

Recent studies highlight the potential role of telemedicine in the early recognition of dementia. For example, patients living in remote and underserved areas can receive specialized assessment through video-based platforms, which overcome geographical barriers [16,104,105]. The use of telemedicine has been related to effective, safe, patient-centered and efficient care for patients with dementia [16,104,105]. A recently developed telemedicine protocol for cognitive disorders in Greece has incorporated the MBI-C during the video sessions for individuals with cognitive complains without dementia [106]. In this context, embedding telehealth modalities within MBI-screening frameworks could enhance equity and inclusion regarding the access to high-quality care.

### 3.8. Dementia Research and Innovation (Objective 7)

Neuroimaging data show that MBI is associated with structural brain changes, which are implicated early in the course of AD [11,30,31,32,33,34,35]. In the Atherosclerosis Risk in Community (ARIC) study (*n* = 1445), MBI was linked to lower gray matter volumes in temporal lobes, including the hippocampus [32]. Affective dysregulation and impulse dyscontrol were linked to reduced gray matter volumes in the inferior temporal lobe and parahippocampal gyrus respectively [32]. MBI as a whole and the emotional dysregulation domain have been related to decreased cortical thickness in the right supramarginal and parahippocampal gyri [107]. In another study in patients with mild stroke, MBI is linked to reduced cortical thickness of the precuneus cortex, a region critically implicated in early AD [108]. Moreover, MRI-based analyses from the NACC cohort showed that MBI, unlike non-MBI NPS, was associated with more widespread AD-pattern neurodegeneration (reduced hippocampal and cortical thickness) and with a more rapid course of cognitive decline [40]. Therefore, structural MRI markers could be used in improving MBI detection and refining risk stratification strategies.

Specific MBI domains have also been linked to distinct structural covariance network patterns in AD. In particular, affective dysregulation has been associated with alterations in the right inferior frontal gyrus network properties, while social inappropriateness and impulse dyscontrol with abnormalities in the structural network of the right posterior cingulate cortex [109]. Apathy has also been associated with alterations in the frontal-executive circuit in individuals with MCI [110], suggesting that neuroimaging biomarkers could give us further insights into the NPS in pre-dementia stages.

Recent neuroimaging evidence links the MBI impulse dyscontrol domain to microstructural abnormalities in AD-vulnerable circuits [111]. Diffusion-tensor magnetic resonance imaging (DTI) evidence has shown that individuals with impulse dyscontrol may show abnormal fornix integrity, as well as altered fronto-occipital, cingulum and uncinate fasciculi diffusivity, patterns characteristic of early AD [111], further supporting its promising value within biomarker-based models.

MBI has been associated with decreased functional connectivity in networks that are disrupted in dementia [112]. In Ontario Neurodegenerative Disease Research Initiative (ONDRI) cohorts, disinhibition and apathy were associated with functional connectivity between the posterior salience network and default mode network in individuals with MCI due to AD [113]. In a study including individuals with normal cognition and amnestic MCI, MBI was related to abnormalities in the functional connectivity of the frontoparietal control network (FPCN), particularly in the domain of affective dysregulation [35]. Complementing these findings, tau pathology has been shown to influence MBI indirectly via altered functional segregation of the salience network from other association networks, underscoring the pivotal role of salience-network integrity in linking AD pathology to early NPS [114]. Collectively, these results highlight potential therapeutic targets, including neuromodulation and circuit-informed behavioral interventions, and advance the translational relevance of MBI in dementia research.

In addition to AD biomarkers, MBI has been associated with metabolic markers, such as insulin, homocysteine and ferritin, implying that a multifaceted pathophysiological basis underlying this condition, paving the way for the better understanding of the neurobiological mechanisms of neurodegeneration at very early stages [79,115].

Furthermore, MBI may index underlying neuropathology or disease progression beyond AD. For instance, in isolated REM sleep behavior disorder (iRBD), a prodromal synucleinopathy, individuals with MBI displayed increased amyloid burden in prefrontal and subcortical regions, despite comparable cognition and glucose metabolism [116]. In accordance, MBI-C could discriminate well the individuals with iRBD and increased risk of phenoconversion to clinically established Lewy body disease (LBD), compared to those with a low risk [117]. In a PET/SPECT study combining amyloid and dopamine-transporter imaging, individuals with both amyloid and putative Lewy body pathology exhibited higher MBI abnormal perception/thought content scores, pointing toward early psychotic features in mixed AD-Lewy body disease [118]. In patients with early-to-mid stage Parkinson’s disease (PD), MBI has also been linked to white matter microstructural alterations [119]. Therefore, it can be speculated that MBI might be an indicator of co-existing amyloid pathology in PD or Lewy body dementia (LBD) [120], reflecting a possibly more rapid disease course, supporting its utility for stratification and biomarker-enriched research designs beyond AD.

Additional research in PD and MBI sheds also more light in the pathophysiology of both conditions. MBI has been associated with worse cognitive performance and temporal lobe atrophy in PD, which has been linked to more rapid cognitive decline [121]. Further functional MRI (fMRI) work shows that MBI corresponds to alterations in large-scale neural networks in PD [122]. During set-shifting tasks, MBI in PD patients has been related to decreased activation in posterior parietal and prefrontal cortices, as well as abnormal deactivation of medial temporal areas [122]. In another study in PD patients, MBI was associated with worse cognitive function, more severe and motor impairment, and altered levels of dopamine transporter (DAT) in the anterior caudate and putamen [123]. In agreement, MBI has been related to disrupted corticostriatal connectivity in PD, especially between the caudate head and cortical areas linked to the default mode network (DMN) and saliency networks (SA), reinforcing the value of MBI as a marker in PD.

Evidence from structural neuroimaging research also supports the prognostic value of MBI for frontotemporal pathology. In a longitudinal MRI study, individuals with MBI who later converted to dementia, predominantly behavioral variant frontotemporal dementia (bvFTD), demonstrated at baseline more frequently focal frontal atrophy, impaired Theory of Mind and executive dysfunction [124]. The combination of these behavioral, cognitive, and structural features could accurately distinguish converters from non-converters, highlighting MBI’s utility for early identification of individuals on a frontotemporal neurodegenerative trajectory.

Emerging neuroimaging evidence also links MBI with cerebrovascular brain changes. In the MEMENTO cohort including older adults with MCI, MBI was associated with greater white matter hyperintensity (WMH) volume, while cognitive performance assessed by MMSE did not [125]. These findings suggest that MBI may serve as a behavioral proxy for underlying small-vessel disease burden, supporting its integration into multimodal biomarker frameworks and future precision-medicine trials.

There is growing recognition that MBI may substantially improve dementia clinical trial efficiency by enriching cohorts with individuals in the true preclinical or prodromal phase. As MBI may precede detectable cognitive impairment, MBI screening can improve signal-to-noise ratios and reduce sample size requirements [99]. The International Clinical Trials in Alzheimer’s Disease (CTAD) Task Force has also recently emphasized MBI as a pivotal construct for advancing clinical trial design in preclinical and prodromal AD [126]. It is recommended that MBI itself may become a direct therapeutic target, advocating for biomarker-confirmed, disease-specific MBI treatment trials [126]. The Task Force proposes pharmacologic and psychotherapeutic interventions tailored to MBI domains, and the MBI-C is highlighted as the optimal instrument for MBI assessment [126]. The combined use of SCD and MBI measurements has also been proposed an attractive opportunity for enriching clinical trial participants’ pools for investigating prevention strategies [120]. These innovations align with the broader research agenda to accelerate therapeutic development and overcome long-standing challenges in trial recruitment and early disease detection.

The MBI-C has been validated across several countries and linguistic contexts, enabling its application in diverse clinical and research settings [127,128,129,130,131,132]. These validation studies provide insights into how NPS may be expressed, perceived and reported in different sociocultural environments. For instance, the psychometric properties of the Greek MBI-C demonstrated in a recent study suggest that strong familial support networks and cultural norms attributing behavioral changes to normal aging might possibly obscure the recognition of subtle symptoms such as decreased motivation or social withdrawal [127]. Such findings highlight the need for culturally sensitive assessment tools, especially for populations under-represented in dementia research. In this way, MBI-C validation studies contribute to improved comparability of findings and greater equity and inclusivity in dementia research.

Interestingly, a short form for the self-assessment of MBI has been recently developed and validated in individuals without dementia, called Mild Behavioral Impairment Scale (MBI-S) consisting of eight items [133]. This scale is more time-efficient than MBI-C, and its use can be considered in cases of time limitations. Another 19-item screening tool for MBI has been developed [134], although its use remains limited.

Therefore, embedding MBI screening into trial recruitment may increase efficiency, improve enrichment for preclinical and prodromal disease, enhance signal-to-noise, and open avenues for novel behavioral biomarker discovery. MBI can also guide precision-prevention strategies and digital phenotyping research, representing a frontier for innovation linking neuroscience, digital health, and prevention policy.

Collectively, the evidence suggests that MBI bridges the gap between individual-level behavioral science and population-level dementia prevention strategies. By integrating MBI across all pillars of the WHO Global Action Plan, health systems may achieve earlier identification, better resource allocation, and more inclusive public health responses to dementia.

## 4. Discussion

Growing evidence positions MBI as the neurobehavioral axis of pre-dementia stage, complementing the traditional neurocognitive axis of MCI that has historically dominated early AD detection. MBI captures later-life emergent and persistent behavioral changes that often precede measurable cognitive decline, reflecting early disruptions in neural circuits vulnerable to AD pathology. As such, MBI represents a promising behavioral marker that may facilitate earlier identification of at-risk individuals, aligning closely with all seven pillars of the WHO Global Action Plan on Dementia.

Appropriate operationalization of MBI is essential for dementia prognostication. Recent evidence demonstrates that definitions requiring persistent symptoms across repeated visits, closer to the ISTAART criteria, identify individuals at substantially higher dementia risk than single-timepoint definitions [135]. While tools like the NPI can approximate MBI in retrospective datasets, the MBI-Checklist remains the only instrument specifically designed to capture symptom persistence and domain-level structure, underscoring its importance for research precision and clinical translation.

The mode of assessment is another critical consideration. Weak-to-moderate concordance between self-reported and informant-rated MBI-C scores highlights the complex nature of symptom awareness, and informant-related factors [136]. Differences in the time spent with the individual and the relationship type (parent, spouse, other relative) may affect NPS recognition and report.

Anosognosia should be also considered, as individuals might not recognize well their impulsivity or irritability, resulting in potential underreporting. The somewhat higher prevalence captured by informant ratings may reflect greater sensitivity to subtle behavioral change [137]. These discrepancies underscore the importance of multi-source assessment frameworks, particularly in community and primary-care settings where objective biomarkers may be unavailable.

A rapidly expanding literature indicates that MBI may provide a clinical window into the earliest biological processes of neurodegeneration. Convergent associations across CSF, plasma, PET imaging, and post-mortem studies demonstrate links between MBI and core AD pathologies, including amyloid and phosphorylated tau. Longitudinal findings using tau, amyloid and NfL biomarkers among individuals with MBΙ further support its potential utility as a dynamic marker of disease progression rather than a static, cross-sectional phenomenon.

Mechanistic work further suggests that MBI may reflect early dysregulation across multiple interconnected pathways, including hypothalamic-pituitary-adrenal (HPA)-axis dysfunction, impaired neurotrophic signaling including the brain-derived neurotrophic factor (BDNF), neuroinflammatory activation such as the kynurenine pathway, epigenetic alterations including imbalance in several micro-RNAs, imbalance in neurotransmitters such as acetylcholine, serotonin, dopamine, norepinephrine and gamma-aminobutyric acid (GABA), synaptic dysfunction, and locus coeruleus dysfunction [4,5,138,139,140]. These multifactorial pathways mirror emerging models of early AD pathogenesis and position MBI as a behavioral expression of preclinical neurobiological vulnerability.

Recent advances also reveal important insights into the genetic architecture of MBI, suggesting that specific MBI domains map onto distinct molecular pathways implicated in AD and other dementias. Associations with APOE and MS4A for affective dysregulation, ZCWPW1, EPHA1, and BIN1 for psychosis-related symptoms, and NME8 for apathy highlight the possibility that MBI domains represent clinically observable phenotypes of underlying genetic susceptibilities [9,51,70,74,141,142,143,144]. Evidence linking MBI to BDNF polymorphisms in PD further suggests that the syndrome may represent a convergent behavioral phenotype across multiple neurodegenerative processes [39]. Studying MBI at the genetic level may therefore accelerate the development of targeted, mechanism-based preventive and therapeutic strategies.

MBI not only correlates with core AD biomarkers but may also capture functional and cognitive vulnerabilities invisible to traditional screening. MBI has been associated with faster decline in attention and working memory, greater functional impairment in instrumental activities of daily living, and poorer health-related quality of life even in cognitively intact individuals. Domain-level differences carry important clinical implications: apathy, affective dysregulation, and impulse dyscontrol may appear particularly predictive of conversion to MCI or dementia, whereas multi-domain MBI yields one of the strongest clinical risk profiles identified to date.

Importantly, the syndrome is modifiable. As shown in clinical studies, multidomain non-pharmacological interventions, including dance-based exercise programs, cognitive stimulation, and empowerment-based psychosocial interventions can reduce MBI symptoms while improving quality of life and cognitive performance. This suggests that incorporating MBI screening into primary care, community-based prevention programs, and telemedicine pathways could potentially enhance early intervention efforts.

Beyond its clinical relevance, MBI offers substantial value at the population and public health levels. It could be integrated into national dementia surveillance systems, digital monitoring platforms, and WHO dementia observatories to improve detection of at-risk individuals. Given its alignment with modifiable risk factors, such as physical activity, social engagement, hearing loss, and psychological resilience, MBI also represents a potential intervention target within risk-reduction strategies. Incorporating MBI into public awareness campaigns, caregiver training programs (e.g., adaptations of WHO’s iSupport), and telemedicine-based models of behavioral monitoring may further strengthen early detection, timely support, and prevention-focused care pathways.

From a policy and implementation perspective, translating MBI-informed approaches into real-world dementia prevention strategies will require careful consideration of contextual, cultural, and system-level factors. Challenges may include limited workforce capacity, variability in health literacy, and competing priorities within primary care, particularly in resource-constrained settings. Task-shifting strategies, whereby MBI screening is conducted by trained non-specialist health workers or integrated into routine primary care assessments, may offer a feasible solution, consistent with WHO recommendations for scalable mental health and dementia care. Cultural adaptation of MBI assessment tools and educational materials is also critical, as the interpretation and reporting of later-life behavioral changes are strongly shaped by sociocultural norms, stigma, and family structures. Without appropriate adaptation, MBI symptoms may be normalized, misattributed, or underreported, reducing the effectiveness of screening initiatives.

In low- and middle-income countries, phased or stepped-care models could facilitate implementation, whereby MBI screening serves as an initial triage step to identify individuals who may benefit most from targeted risk-reduction interventions, further assessment, or specialist referral. Such models are particularly relevant in health systems with limited specialist availability, as they allow prioritization of care based on behavioral risk rather than access to advanced diagnostics. In settings where access to biomarker testing is limited, MBI assessment may function as a pragmatic behavioral entry point into dementia risk stratification, enabling more efficient allocation of specialized diagnostic resources. Importantly, embedding MBI screening within existing primary care, community health, or chronic disease management programs may enhance feasibility, sustainability, and equity, in line with the WHO Global Action Plan’s emphasis on scalable and inclusive dementia prevention strategies. Context-sensitive implementation, including adaptation to local care pathways, workforce capacity, and sociocultural perceptions of behavioral change, will be essential to ensure that MBI-based approaches reduce rather than exacerbate existing health disparities.

Embedding MBI within existing public health infrastructures, such as chronic disease management programs, aging services, or community-based brain health initiatives, may enhance sustainability and cost-effectiveness while minimizing fragmentation of care. Aggregated MBI data collected at the population level could inform public health surveillance of behavioral risk states, supporting more responsive and data-driven dementia prevention strategies. Implementation research evaluating acceptability, feasibility, and equity of MBI-based strategies across diverse settings will be essential to inform policy decisions and ensure that early detection efforts translate into meaningful public health impact rather than widening existing disparities. Despite the rapidly growing evidence base, several limitations should be acknowledged. While the existing literature provides converging support for the relevance of MBI across the dementia continuum, the overall quality and structure of the evidence remain heterogeneous. First, substantial heterogeneity exists in how MBI is operationalized across studies, with many relying on retrospective NPI-based algorithms that do not fully capture the ISTAART-AA chronicity and domain-level criteria. This methodological inconsistency complicates cross-study comparisons and may partly explain variability in reported effect sizes.

Second, the majority of biomarker and neuroimaging studies derive from highly selected samples, most notably the ADNI cohort, which may not be representative of the general population or of under-studied groups, including individuals from low- and middle-income countries. Third, many available datasets rely on informant ratings, and informant-related factors (e.g., relationship type, mood, age) may influence symptom reporting, potentially confounding associations with biological markers. Fourth, the cross-sectional nature of several biomarker studies limits causal inference regarding temporal ordering between emergent behavioral symptoms and neuropathological changes. Finally, although randomized interventions targeting MBI are emerging, the evidence base for treatment responsiveness remains limited.

Importantly, there is a striking underrepresentation of low- and middle-income countries in MBI research, despite these regions bearing a rapidly increasing dementia burden. Differences in referral pathways, access to neuropsychological evaluation, and cultural interpretations of behavioral change may substantially influence the feasibility and clinical impact of MBI-based screening. This gap limits insight into how MBI assessment performs across diverse healthcare systems, cultural contexts, and levels of specialist availability, underscoring the need for globally inclusive, methodologically harmonized studies.

An additional limitation of the current evidence base relates to the relatively limited integration of MBI and MCI within unified conceptual and analytic frameworks. Although MBI and MCI frequently co-occur and are each independently associated with increased dementia risk, relatively few studies have systematically examined their combined trajectories, interactions, or additive prognostic value. As a result, it remains unclear whether specific MBI domains confer incremental risk beyond cognitive impairment, whether behavioral and cognitive changes follow distinct temporal patterns, or how these constructs may jointly inform risk stratification and prevention strategies. Addressing these gaps will require longitudinal studies explicitly designed to model MBI–MCI interactions to support the translation into clinical and public-health settings.

Overall, while MBI is consistently associated with dementia risk and biological markers across multiple study designs, causal relationships should be tested in future interventional and mechanistic studies. Future research should prioritize the development of harmonized, standardized MBI assessment protocols across clinical and research settings, ensuring the routine use of the MBI-C or possibly the shorter form of MBI-S, and incorporating appropriate repeated measurements to capture symptom persistence.

Large-scale, longitudinal, multimodal studies integrating behavior, cognition, genetics, digital phenotyping, and fluid or neuroimaging biomarkers are needed to delineate the mechanistic pathways linking MBI to disease progression and to determine whether MBI is merely a clinical correlate or an active driver of accelerated neurodegeneration. Greater representation of diverse populations, culturally distinct groups, and individuals with limited access to specialty care is essential to enhance global generalizability and equity in early dementia detection.

There is also substantial opportunity for innovation in digital and remote monitoring. Passive sensing technologies, speech analysis, ecological momentary assessment, and wearable devices could capture subtle behavioral trajectories long before clinical symptoms become apparent. Interventional research should expand beyond proof-of-concept trials to rigorously test scalable, multidomain lifestyle, psychosocial, and digital therapeutics tailored to specific MBI domains.

Finally, integrating MBI into precision-prevention frameworks and clinical trial enrichment strategies may accelerate the discovery of mechanism-based treatments and facilitate earlier, more effective interventions across the AD continuum.

## 5. Conclusions

MBI may represent a pivotal shift in the conceptualization of pre-dementia states, reframing later-life emergent NPS as early, clinically meaningful indicators of neurodegenerative disease. Across clinical, biomarker, neuroimaging, genetic, and epidemiological studies, MBI consistently aligns with the earliest detectable signatures of AD and other dementias, often predating measurable cognitive decline.

The MBI framework dovetails with all seven objectives of the WHO Global Action Plan, offering a potential scalable, low-cost, and globally adaptable tool to strengthen prevention, early detection, equitable care, and research innovation. Integrating MBI assessment into routine clinical workflows, primary care pathways, digital monitoring systems, and precision-prevention research could accelerate the identification of at-risk individuals and improve the timeliness and effectiveness of interventions.

At the same time, incorporating caregiver education and culturally adapted support resources is essential for reducing burden and promoting well-being across diverse settings. Collectively, the evidence underscores MBI as a powerful, yet underutilized, behavioral lens into early neurodegeneration. Advancing its implementation across health systems and research infrastructures holds substantial promise for transforming how the global community detects, understands, and ultimately prevents dementia in the coming decades.

## Figures and Tables

**Table 1:** Mapping MBI to the WHO Global Action Plan on Dementia (2017–2025, extended to 2031).

WHO Global Action Plan Objective	Relevance of MBI	Key Supporting Evidence	Implications for Policy and Practice	References
1. Dementia as a public health priority	MBI is an early marker of cognitive decline Its high prevalence and prognostic value justify its recognition as a population-level health priority	MBI is associated with faster progression to dementia compared to non-MBI NPS Global Action Plan: need for early detection and risk stratification	Incorporating MBI into national dementia strategies may reinforce early identification, improves prioritization of early stages, aligned with WHO’s call for population-level surveillance of dementia risk states	[7,8,9,10,11,12,13,14,15]
2. Dementia awareness and friendliness	MBI reframes later-life emergent NPS as early indicators of neurodegenerative disease rather than primary psychiatric disorders This conceptual shift aligns with the WHO emphasis on reducing stigma and promoting dementia literacy	Clinicians and community stakeholders may misattribute NPS to depression or anxiety alone WHO: stigma and misunderstanding may delay help-seeking and worsen outcomes	Integrating MBI into public campaigns, professional training, and dementia-friendly initiatives may reduce stigma, promote early recognition, and support earlier access to care	[16]
3. Dementia risk reduction	MBI is associated with brain–behavior changes linked to AD pathology and vascular risk factors, fitting WHO’s emphasis on risk reduction and brain health promotion	MBI may predict AD pathology years later Diabetes, physical inactivity, and poor social support are associated with higher MBI burden WHO highlights lifestyle-based dementia prevention: vascular health, physical activity, & social engagement	MBI can serve as a behavior-first triage tool for identifying individuals who may benefit most from risk-reduction programs MBI may increase the reach and efficiency of primary prevention	[17,18,19,20,21]
4. Diagnosis, treatment, care and support	MBI may provide a structured framework for identifying preclinical and prodromal neurodegenerative changes, consistent with WHO calls for earlier diagnosis and better continuity of care	MBI correlates with AD biomarkers (amyloid, p-tau), tau-PET patterns, plasma NfL MBI clusters may predict differential cognitive trajectories MBI is associated with early IADL decline and lower HRQoL WHO: need for earlier, equitable diagnosis and improved care pathways	MBI may enhance timely referrals, improve stratification in memory clinics, and support mechanism-informed care planning Behavioral screening combined with biomarkers aligns with WHO recommendations for holistic care models	[22,23,24,25,26]
5. Support for dementia carers	MBI is associated with caregiver burden even in case of minimal cognitive symptoms, highlighting early carer needs overlooked in standard dementia frameworks	MBI is associated with caregiver burden WHO: emphasizes caregiver training and support	Carer-support interventions such as WHO’s iSupport could be expanded to include modules for MBI education, management, and early recognition. Cultural adaptation is essential across countries	[27]
6. Information systems for dementia	MBI can be a scalable, low-cost marker suitable for integration into national surveillance and WHO’s Global Dementia Observatory	The MBI-C may facilitate standardized, remote, population-level data capture WHO calls for digital health information systems and behavioral data collection	Incorporating MBI into electronic health records, registries, and national dementia observatories may enhance early-stage surveillance MBI-C is suitable for digital monitoring, mobile health tools, and automated behavioral change detection, supporting real-time risk tracking	[28,29]
7. Dementia research and innovation	MBI may offer a novel behavioral phenotype linked to neuropathological, neuroimaging, biomarker, and digital biomarker signatures, an ideal target for research and preclinical and prodromal trial enrichment	MBI correlates with amyloid burden, WMH burden, tau-PET, frontotemporal atrophy, plasma NfL trajectories, impaired frontoparietal networks (MRI/fMRI), & altered task-related activation in PD-MBI WHO: need to double research output and develop early detection markers	Embedding MBI screening into trial recruitment may increase efficiency, enrich, enhance signal-to-noise, and open avenues for biomarker discovery MBI can guide precision-prevention strategies and digital phenotyping research	[11,30,31,32,33,34,35]

## Data Availability

No new data were created or analyzed in this study. Data sharing is not applicable to this article.
